# Preharvest potassium silicate treatments influence the morphological appearance and enhance nutritional composition of almond kernels

**DOI:** 10.1002/jsfa.70392

**Published:** 2025-12-16

**Authors:** Vicente Serna‐Escolano, María Á. Botella, Alessio Allegra, Pedro J. Zapata, María J. Giménez

**Affiliations:** ^1^ Institute for Agro‐food and Agro‐environmental Research and Innovation (CIAGRO), EPSO, Miguel Hernández University (UMH) Orihuela Spain; ^2^ Department of Agricultural, Food and Forest Sciences University of Palermo Palermo Italy

**Keywords:** fatty acids, nutrients, phenolics, *Prunus dulcis*, total antioxidant activity

## Abstract

**BACKGROUND:**

The almond industry requires new strategies to improve almond kernel quality. The use of biostimulants such as potassium silicate (KSi) is an eco‐friendly and non‐polluting alternative. The aim of this work was to investigate the preharvest application of KSi solutions at 2 and 20 mmol L^−1^ to ‘Peraleja’ almond trees during two consecutive seasons to elucidate the effect on almond kernel quality at harvest.

**RESULTS:**

The fresh weight, length and width of almond kernels treated with 20 mmol L^−1^ KSi increased by 27%, 31% and 8%, respectively, compared to the controls. Furthermore, almonds treated with 20 mmol L^−1^ KSi had a more elongated morphological appearance, while with 2 mmol L^−1^ KSi almonds were more spherical. Regarding nutrients, phosphorus and potassium levels increased in almonds treated with 2 mmol L^−1^ KSi, while magnesium content decreased with both KSi treatments. Total antioxidant activity and total phenolic content increased in almonds treated with 20 mmol L^−1^ KSi. In addition, significant differences in fatty acid levels were observed, particularly in oleic and linoleic acids. On average, the oleic–linoleic acid ratio of almonds treated with 2 mmol L^−1^ KSi was 28% higher than those treated with 20 mmol L^−1^ KSi and the controls.

**CONCLUSION:**

These results demonstrate that the preharvest application of KSi is a valuable tool, as growers can use different KSi concentrations to target specific improvements in almond kernel quality, including physical attributes and nutritional composition. © 2025 The Author(s). *Journal of the Science of Food and Agriculture* published by John Wiley & Sons Ltd on behalf of Society of Chemical Industry.

## INTRODUCTION

The almond (*Prunus dulcis* Mill. D.A. Webb) is one of the most widely produced nuts in the world, with an annual production of 3 Mt in 2024. The most important producer is USA, with 1.8 Mt, followed by the countries of the Mediterranean region.^1^ The almond has an outer hard shell that protects the edible seed (kernel), which can be consumed raw, sliced, roasted, or combined with other nuts. The kernel can also be used to make other products known as marzipan and nougat.^2^ In the almond industry, quality is primarily defined by physical parameters such as size and shape. However, considering kernel composition is a key to developing higher‐quality products with enhanced nutritional value for consumers. Almonds are therefore considered a healthy product due to their functional composition, which has also been linked to beneficial effects on cardiometabolic diseases such as obesity, hypertension, diabetes mellitus and metabolic syndrome.^3^ Almonds are a significant source of lipids, accounting for 50% or more of the dry weight. The high levels of unsaturated fatty acids (UFA), particularly monounsaturated fatty acids (oleic acid), contribute to increase the phytonutrient value of almonds because they do not contribute to cholesterol formation in humans.^4^ Furthermore, almond kernels contain high levels of phenolic compounds with antioxidant properties, the most important of which is α‐tocopherol (vitamin E). Tocopherols are well known for inhibiting lipid peroxidation and protecting polyunsaturated fatty acids against the activity of free radicals.^5^ Other important almond constituents are proteins (globulins), carbohydrates and essential minerals, representing 20%, 10% and 4% of the dry weight, respectively.^6^

Generally recognised as safe (GRAS) salts have been widely used in postharvest treatments, either by direct dipping or as part of edible coatings. These treatments delay ripening and senescence, reduce fungal activity and extend the shelf life of fruit.^7^ However, the use of inorganic salts has emerged as a novel method for alleviating plant stress and increasing the nutritional quality of fruit and vegetables. In this sense, potassium silicate (KSi) is a GRAS salt, source of potassium (K) and silicon (Si) for agricultural management. Although Si levels are not critical for plants, its presence has been associated with an improved response to abiotic stress and the maintenance of cellular integrity by increasing the accumulation of phenolic polymers.^8^ Previous results have shown that Si treatments improve the antioxidant system, increase proline accumulation, regulate polyamine biosynthesis, enhance the activity of enzymes related to the photosynthesis and determine the accumulation of chlorophylls.^9^, ^10^ Furthermore, K is an essential macronutrient that affects photosynthetic rate, enhances carbon dioxide assimilation and plays a significant role in facilitating the translocation of carbohydrates and minerals from roots to leaves and fruits.^11^ Combined preharvest KSi treatments have been associated with an increase in the total antioxidant activity and total phenolic content in ‘Navel’ and ‘Valencia’ oranges,^12^ improved plant growth, yield and fruit quality in sweet peppers^13^ and reduced oxidative stress, thereby contributing to the maintenance of membrane stability in Cape gooseberry fruits.^14^

In Spain, most almond crops are cultivated in the arid and semi‐arid areas of the Mediterranean region, where rainfall is scarce and evaporation rates are high. These factors reduce the quality of almonds by affecting visual and chemical parameters.^15^ Therefore, the aim of this study was to evaluate the impact of preharvest KSi treatments on the morphological, physicochemical and functional quality of ‘Peraleja’ almond kernels during two consecutive seasons.

## MATERIAL AND METHODS

### Experimental design

The experiment was carried out in non‐irrigated commercial fields located in Orihuela, Alicante, Spain (38° 00′ 24.3" N, 0° 55′ 35.0" W), which had an average temperature of 18.4 °C and rainfall of 280 mm in the 2023 season, and an average temperature of 17.9 °C and rainfall of 310 mm in the 2024 season. For each season, three blocks of three 10‐year‐old ‘Peraleja’ almond trees were randomly selected and treated with potassium silicate (KSi) at concentrations of 2 and 20 mmol L^−1^. These concentrations were selected according to the results previously published by Serna‐Escolano *et al*.^12^ All salt treatments were prepared by diluting KSi (Sigma‐Aldrich, Madrid, Spain) in distilled water, adding 0.5% Tween 20 as a surfactant. Control trees were treated with 0.5% Tween 20 dissolved in an aqueous solution. The almond trees were treated with 5 L of the solution via foliar spraying using a mechanical system. These treatments were applied three times: the first application was conducted when the final almonds were setting on the tree in May and June of the 2023 and 2024 seasons, respectively; the second application was conducted 1 month later; and the third application took place 3 days before harvest in order to maximize the stimulation of the almond kernels. The almonds were harvested in July 2023 and August 2024, once they had reached the stage of commercial ripeness required by the market. The almonds were then exposed to sunlight horizontally until their moisture content was below 5%. Subsequently, the in‐shell almonds were transported to the laboratory for subsequent analytical determinations.

### Morphological and physical characterization

The weight, length, width and thickness of 50 almonds from each treatment were measured to determine their shape and size. A scale (AG204, Mettler Toledo, Barcelona, Spain) and a digital calliper (500‐164‐30, Mitutoyo, Kawasaki, Japan) were used for the measurements. The length‐to‐width ratio (L/W) was calculated, and the following equations were used to calculate several geometric factors, such as geometric mean diameter (GMD), sphericity (∅), and surface area (*S*):

(1) $$\mathrm{GMD}\;\left(\mathrm{mm}\right)={\left(\text{Length}\times \text{Width}\times \text{Thickness}\right)}^{\frac{1}{3}}$$

(2) $$\emptyset \left(\%\right)=\frac{\mathrm{GMD}}{\text{Length}}\times 100$$

(3) $$S\;\left({\mathrm{cm}}^{2}\right)=\pi \times {\mathrm{GMD}}^{2}\times 10^{-2}$$

Furthermore, the texture of 50 almonds per treatment was determined using a texture analyser (model TA‐XT2i, Stable Micro Systems, Godalming, UK) fitted with a 30 kg load cell and a Volodkevich Bite Jaw HDP/VB probe. The trigger was set at 15 g, with a test speed of 1 mm s^−1^ over a specified distance of 3 mm. The obtained parameters were hardness (N) and fracturability (mm).

### Moisture content

The moisture content of six samples, each containing ten almond kernels, was determined by placing them in a pre‐weighed aluminium tray in an oven (Digitron C2005141, Selecta, Abrera, Barcelona, Spain) and drying them at 105 °C until they reached a constant weight (adapted from AOAC official method 925.40). The moisture content was calculated based on the initial and final sample weights using Eqn (4), and the results are expressed as a percentage:

(4) $$\text{Moisture}\;\left(\%\right)=\frac{\left(W_{i}-W_{f}\right)}{\left(W_{i}-W_{1}\right)}\times 100$$

where *W*_i_ and *W*_f_ are the weights of the moisture trays containing the almond samples before (initial) and after (final) drying, respectively, and *W*_1_ is the weight of the empty aluminium tray.

### Mineral composition

The mineral content was determined by digesting 0.5 g of each of the six samples (each containing ten almond kernels) per treatment. The ground almonds were placed in a muffle furnace (Hobersal, Barcelona, Spain) at a temperature of 650 °C for 6 h. Additionally, 1 mL of 6 mol L^−1^ HCl was added to the obtained ash and transferred to a 25 mL volumetric flask. Dilutions of 1:25 and 1:10 were prepared using ultrahigh‐purity deionized water, after which the samples were stored at 4 °C until analysis. The determination of the macro (N, P, K, Ca, Mg, Na and S) and micro (Fe, Mn, Cu, Zn and B) element content of the previously mineralized samples was determined using an atomic absorption–emission spectrometer (Solar 969, Unicam Ltd, Cambridge, UK). Potassium and sodium were analysed using atomic emission, while the other elements were analysed by atomic absorption. The instruments were calibrated using certified standards. Results were expressed as grams per kilogram.

### Total phenolics and total antioxidant activity

The total phenolic content (TPC) of the almond kernels was determined by homogenizing 0.5 g of the sample powder (six samples per treatment, each with ten almond kernels) in a solution of water and methanol (ratio 2:8) containing 2 mmol L^−1^ NaF, which was used to inactivate polyphenol oxidase activity. The homogenate was then centrifuged at 15 000 × *g* for 15 min at 4 °C, after which the TPC was quantified in duplicate in the supernatant using Folin–Ciocâlteu reagent, as previously described by Serna‐Escolano *et al*.^16^ The results are the mean ± standard error (SE) of six replicates, expressed as grams of gallic acid equivalents per kilogram dry weight (DW). To determine total antioxidant activity (TAA), 0.5 g of almond kernels were homogenized in 10 mL of 50 mmol L^−1^ potassium phosphate buffer solution (pH 7) and ethyl acetate (2:1, v/v). The resulting extracts were centrifuged at 10 000 × *g* for 12 min at 4 °C. Antioxidant activity was then measured in both the hydrophobic and hydrophilic phases using the ABTS‐peroxidase system, as previously described by Serna‐Escolano *et al*.^17^ TAA was determined by summing the values from both phases. Results are expressed as the mean ± SE of six samples, and are given in grams of Trolox equivalents per kilogram DW.

### Fatty acid profile

Fatty acid methyl esters (FAMEs) were prepared using *in situ* methylation with some modifications, and analysed according to the method described by Lipan *et al*.^18^ Ground almond (40 mg; six samples per treatment) was saponified with 100 μL dichloromethane (CH_2_Cl_2_) and 1 mL of a sodium methoxide solution, then refluxed for 10 min at 90 °C. Then, 1 mL BF_3_ methanolic solution was added, followed by a 30 min rest in the dark to allow the reaction to occur. Finally, the FAMEs were extracted from the mixture using 1.5 mL hexane and separated using a gas chromatograph (GC17A, Shimadzu, Kyoto, Japan) coupled with a flame ionization detector and a DB‐23 capillary column (Agilent Technologies, Santa Clara, CA, USA). The helium carrier gas flow rate was 1.1 mL min^−1^ and 35 mL min^−1^ at the make‐up point. The injector temperature was 240 °C and the detector temperature was 260 °C. The injection volume was 0.8 μL (split ratio 1:20). The temperature programme was as follows: an initial temperature of 100 °C was held for 1 min; a temperature gradient of 3 °C min^−1^ was applied until 220 °C; then a gradient of 5 °C min^−1^ was applied until 245 °C; finally, the temperature was held at 245 °C for 1 min. FAME peaks were identified by comparing their retention times with those of the Supelco MIX‐37 standard (Sigma‐Aldrich, St Louis, MO, USA). The results were expressed as grams per kilogram using methyl nonadecanoate as the internal standard.

### Statistical analysis

The data for each almond kernel sample were the mean ± SE of six samples per treatment, which were analysed statistically by analysis of variance (ANOVA) with a multiple‐range test (Tukey's test), in order to determine any significant differences between the samples (*P* < 0.05). These analyses were performed using SPSS version 22 (IBM Corp., Armonk, NY, USA).

## RESULTS

The preharvest KSi treatments affected most morphological and physical parameters of almond seeds except fracturability and moisture content (Table 1). The fresh weight results for the 2023 and 2024 seasons were higher in almonds treated with 20 mmol L^−1^ KSi than in the controls, with an increase of 27% and 21%, respectively. However, no effect of 2 mmol L^−1^ KSi was observed on fresh weight. For hardness and kernel length, treatments with 20 mmol L^−1^ KSi showed the highest values in both seasons. With regard to kernel width, it was significantly (*P* < 0.05) increased by KSi treatments in the 2023 season, while no significant (*P* > 0.05) differences were observed in 2024. The highest values of thickness were obtained in almonds treated with 20 mmol L^−1^ KSi compared to those treated with 2 mmol L^−1^ KSi and the controls in the 2024 season, while no significant (*P* > 0.05) differences were observed in 2023. The samples showed differences in L/W, ranging from 1.49 to 1.81 and from 1.92 to 2.16 in the 2023 and 2024 seasons, respectively, with the highest values found in almonds treated with 20 mmol L^−1^ KSi. The treatments significantly affected (*P* > 0.05) sphericity in the 2023 season, which was higher with 2 mmol L^−1^ KSi and the control treatments than with 20 mmol L^−1^ KSi. However, there was no effect on this parameter in the 2024 season. The highest values for GMD and surface area were found in almonds treated with 20 mmol L^−1^ KSi compared to those treated with 2 mmol L^−1^ KSi and the control in both seasons. Significant differences (*P* < 0.05) were observed in all morphological and physical parameters except moisture content, hardness, fracturability and thickness when the two harvest seasons were compared. The highest values of sphericity were found in 2023, while fresh weight, length, width, L/W, GMD and surface area were higher in 2024.

**Table 1:** Effect of preharvest treatments with potassium silicate (KSi) on morphological parameters and physical properties of almond kernels

	2023				2024				ANOVA	2023	2024
	ANOVA	Control	KSi 2 mmol L^−1^	KSi 20 mmol L^−1^	ANOVA	Control	KSi 2 mmol L^−1^	KSi 20 mmol L^−1^			
Fresh weight (g)	**	0.70b	0.75b	0.89a	**	0.81b	0.85b	0.98a	**	0.78	0.88
Moisture (%)	NS	4.33	3.98	4.15	NS	4.25	4.31	4.16	NS	4.15	4.24
Hardness (N)	***	57.04b	60.68b	67.32a	**	61.04b	61.88b	73.40a	NS	61.72	65.44
Fracturability (mm)	NS	1.67	1.70	1.72	NS	1.61	1.65	1.70	NS	1.70	1.65
Length (mm)	***	16.04c	18.94b	21.04a	**	23.35b	23.65b	24.87a	***	18.68	23.98
Width (mm)	***	10.85b	11.80a	11.63a	NS	12.25	12.30	11.73	**	11.42	12.09
Thickness (mm)	NS	6.79	6.76	7.21	***	6.23b	6.28b	7.87a	NS	6.92	6.87
L/W	***	1.49c	1.61b	1.81a	**	1.92b	1.93b	2.16a	**	1.63	2.00
GMD (mm)	***	10.53c	11.42b	12.02a	***	12.09b	12.19b	13.14a	**	11.32	12.48
Sphericity (%)	***	65.99a	60.55b	57.58c	NS	52.07	51.80	53.11	***	61.37	52.33
Surface area (cm^2^)	***	3.50c	4.11b	4.55a	***	4.60b	4.68b	5.45a	**	4.05	4.91

NS, not significant at *P* < 0.05; asterisks indicate significance at *P < 0.05, **P < 0.01 and ***P < 0.001. Values (mean of *n* = 5 samples) followed by the same letter within the same row and season were not significantly different (*P* < 0.05), according to Tukey's least significant difference test.

The results of the mineral content of almonds treated with KSi are shown in Table 2. Regarding macronutrients, KSi application affected P, K and Mg concentration in both seasons, while no effect on Ca concentration was observed. P concentration was not changed with 20 mmol L^−1^ KSi but increased significantly by 15% (2023) and 14% (2024) with 2 mmol L^−1^ KSi. However, K content increased with both KSi levels, by 19% (2023) and 28% (2024) with 2 mmol L^−1^ KSi, and by 10% with 20 mmol L^−1^ KSi in both seasons. The concentration of Mg decreased in almonds under both KSi treatments; this effect was more pronounced in the 2024 season, with decreases of 25% (2 mmol L^−1^ KSi) and 37% (20 mmol L^−1^ KSi) compared to 17% (2 mmol L^−1^ KSi) and 12% (20 mmol L^−1^ KSi) in the 2023 season. The concentration of most micronutrients was not affected by KSi application, except for B, which was significantly (*P* > 0.05) increased in 2023 and 2024, with the highest B concentration found in almonds treated with 2 mmol L^−1^ KSi. Significant (*P* < 0.05) differences were observed in the mineral content of the almonds when comparing the two seasons. In this sense, the highest levels of Ca, S, Fe and B were found in 2023, while K and Mn were higher in 2024.

**Table 2:** Effect of preharvest treatments with potassium silicate (KSi) on macro‐ and micronutrients of almond kernels

Nutrient (g Kg^−1^)	2023				2024				ANOVA	2023	2024
	ANOVA	Control	KSi 2 mmol L^−1^	KSi 20 mmol L^−1^	ANOVA	Control	KSi 2 mmol L^−1^	KSi 20 mmol L^−1^			
N	NS	0.0037	0.0036	0.0038	NS	0.0034	0.0036	0.0038	NS	0.0037	0.0035
P	*	5.5589b	6.4158a	5.4162b	*	6.1999b	6.9768a	6.0384b	NS	5.7969	6.3784
K	**	6.3342c	7.5582a	6.9375b	**	7.4796c	9.5676a	8.1943b	***	6.9433	8.4139
Ca	NS	3.3864	3.6516	3.5598	NS	2.7336	2.7744	2.7438	***	3.5326	2.7506
Mg	**	3.2129a	2.6724b	2.7438b	***	3.3252a	2.4989b	2.1114b	NS	2.8764	2.6452
Na	NS	0.0357	0.0336	0.0326	NS	0.0318	0.0333	0.0308	NS	0.0339	0.0319
S	NS	2.0604	1.9992	2.2236	NS	1.4382	1.5402	1.4178	***	2.0944	1.4654
Fe	NS	0.0384	0.0375	0.0403	NS	0.0277	0.0293	0.0267	***	0.0387	0.0279
Mn	NS	0.0088	0.0093	0.0093	NS	0.0128	0.0132	0.0124	***	0.0091	0.0128
Cu	NS	0.0139	0.0138	0.0147	NS	0.0132	0.0135	0.0131	NS	0.0141	0.0133
Zn	NS	0.0312	0.0342	0.0337	NS	0.0326	0.0339	0.0331	NS	0.0332	0.0332
B	**	0.0171b	0.0247a	0.0217ab	***	0.0128c	0.0182a	0.0173b	**	0.0211	0.0161

NS, not significant at *P* < 0.05; asterisks indicate significance at *P < 0.05, **P < 0.01, and ***P < 0.001. Values (*n* = 5) followed by the same letter within the same row and season were not significantly different (*P* < 0.05), according to Tukey's least significant difference test; N, nitrogen; P, phosphorus; K, potassium; Ca, calcium; Mg, magnesium; Na, sodium; S, sulfur; Fe, iron; Mn, manganese; Cu, copper; Z, zinc; B boron.

The TAA and TPC in the control treatment were higher in the second season (2024) than in the first one (2023). However, this effect was only significant for TPC and not for TAA. The application of KSi treatment on antioxidant capacity (TAA and TPC) had a significant effect at the highest concentration of KSi (20 mmol L^−1^), but no effect was observed at 2 mmol L^−1^ concentration (Table 3). In almonds treated with 20 mmol L^−1^ KSi, an increase of approximately 2.5‐ and 1.5‐fold was observed in 2023 and 2024, respectively, compared to the control. In the 2023 season, the application of 20 mmol L^−1^ KSi increased the TPC value in almonds threefold compared to the control, while in 2024 this effect was less pronounced, with an increase of approximately 10%. The results also showed a slight effect of the harvest season on TPC and no significant (*P* > 0.05) differences for TAA. The interaction between harvest season and treatments was significant (*P* < 0.05) for both TAA and TPC parameters. However, the significance level for TAA was mainly related to the effect of the treatments, whereas for TPC it depended on the combination of both variables.

**Table 3:** Effect of preharvest treatments with potassium silicate (KSi) on the total antioxidant activity (TAA) and total phenolic content (TPC) of almond kernels

	TAA (g kg^−1^)	TPC (g kg^−1^)
*ANOVA*		
Treatment (*T*)	***	***
Harvest season (*H*)	NS	*
*H* × *T*	**	***
*Tukey's multiple range test*		
2023		
Control	1.73b	1.22b
KSi 2 mmol L^−1^	1.74b	1.18b
KSi 20 mmol L^−1^	4.01a	3.76a
2024		
Control	2.15b	1.70b
KSi 2 mmol L^−1^	2.29b	1.70b
KSi 20 mmol L^−1^	3.38a	1.88a

NS, not significant at *P* < 0.05; asterisks indicate significance at *P < 0.05, **P < 0.01 and ***P < 0.001. Values (mean of *n* = 5 samples) followed by the same letter within the same row and season were not significantly different (*P* < 0.05), according to Tukey's least significant difference test.

In the present study, 14 fatty acids were identified and quantified in all treatments, as shown in Table 4. Regarding saturated fatty acids (SFA), palmitic and stearic acids were the most significant. In this sense, palmitic acid increased by approximately 13% in almonds treated with 20 mmol L^−1^ KSi compared to the controls in both seasons, whereas application of 2 mmol L^−1^ KSi had no effect on palmitic acid. The stearic acid content in almonds treated with 20 mmol L^−1^ KSi, increased threefold and 2.5‐fold in the 2023 and 2024 seasons, respectively, compared to the controls. In this case, the treatment with 2 mmol L^−1^ KSi resulted in a slightly higher concentration of stearic acid in the 2023 season. The lipid content of almonds is mainly composed of monounsaturated (MUFA) and polyunsaturated fatty acids (PUFA), with oleic and linoleic acids being the most abundant. It is noteworthy that in most cases the concentration of the different fatty acids was higher in the 2023 season than in the 2024 season. Oleic acid increased significantly as a consequence of both KSi treatments by 10% in 2023, and by 14.3% and 28% in 2024 with 0.1% and 20 mmol L^−1^ KSi, respectively. In addition, linoleic acid content was also increased in almonds treated with 20 mmol L^−1^ KSi in both seasons, whereas it was reduced in almonds treated with 2 mmol L^−1^ KSi in 2023.

**Table 4:** Effect of preharvest treatments with potassium silicate (KSi) on fatty acid composition of almond kernels

Fatty acid (g kg^−1^)	2023				2024				ANOVA	2023	2024
	ANOVA	Control	KSi 2 mmol L^−1^	KSi 20 mmol L^−1^	ANOVA	Control	KSi 2 mmol L^−1^	KSi 20 mmol L^−1^			
C14:0	NS	0.15	0.13	0.15	NS	0.16	0.15	0.15	NS	0.14	0.15
C16:0	**	26.36b	26.41b	29.08a	**	24.14b	26.94ab	28.09a	NS	27.29	26.39
C16:1	*	1.66b	1.85a	1.77ab	NS	2.10	1.69	2.09	NS	1.76	1.96
C17:0	***	0.27c	0.31b	0.38a	*	0.19b	0.18ab	0.23a	***	0.32	0.20
C17:1	*	0.46a	0.38b	0.43ab	NS	0.46	0.38	0.46	NS	0.43	0.43
C18:0	***	5.10c	6.45b	16.60a	***	7.05b	6.11b	17.91a	NS	9.38	10.36
C18:1n9	***	399.39b	442.64a	442.11a	**	268.14c	306.43b	344.32a	***	428.04	306.29
C18:1n7	NS	1.08	1.13	1.11	***	0.8b	0.8b	1.20a	NS	1.11	0.93
C18:2n6	**	105.97b	91.33c	133.77a	***	71.54b	61.95b	121.28a	*	110.36	84.92
C18:3n3	NS	0.30	0.33	0.35	NS	0.40	0.34	0.46	NS	0.33	0.40
C20:0	NS	0.54	0.63	0.58	***	0.33b	0.27b	0.63a	*	0.58	0.41
C22:0	NS	0.14	0.16	0.18	***	0.09c	0.14b	0.18a	NS	0.16	0.14
C22:6n3	***	0.45c	0.78b	1.50a	***	0.26b	1.37a	1.30a	NS	0.91	0.98
C23:0	NS	0.29	0.35	0.34	NS	0.32	0.34	0.38	NS	0.33	0.35
O/L	**	3.77b	4.85a	3.31b	**	3.80b	4.98a	2.89c	NS	3.98	3.91
∑SFA	***	32.57c	34.13b	47.11a	***	32.29b	34.13b	47.57a	NS	37.94	38.00
∑MUFA	***	402.59b	445.99a	445.42a	***	270.77c	308.57b	346.99a	***	431.34	308.78
∑PUFA	***	106.44b	92.16c	135.29a	***	72.19b	63.65b	123.05a	**	111.30	86.30
PUFA/SFA	NS	3.27	2.70	2.88	**	2.24ab	1.88b	2.58a	*	2.95	2.23
PUFA/MUFA	*	0.26b	0.21b	0.30a	**	0.27b	0.21b	0.36a	NS	0.26	0.28
(MUFA+PUFA)/SFA	**	15.63a	15.77a	12.35b	*	10.66a	10.97a	9.87b	***	14.58	10.50
AI	NS	0.05	0.05	0.05	NS	0.07	0.07	0.06	NS	0.05	0.07
TI	NS	0.13	0.13	0.15	NS	0.18	0.18	0.19	*	0.14	0.18

NS, not significant at *P* < 0.05; asterisks indicate significance at *P < 0.05, **P < 0.01 and ***P < 0.001. Values (*n* = 5) followed by the same letter within the same row and season were not significantly different (*P* < 0.05), according to Tukey's least significant difference test; C14:0, myristic; C16:0, palmitic; C16:1, palmitoleic; C17:0, margaric; C17:1, *cis*‐heptadecenoic; C18:0, stearic; C18:1n9, oleic; C18:1n7, *cis*‐vaccenic; C18:2n6, linoleic; C18:3n3, α‐Linolenic; C20:0, arachidic; C22:0, behenic; C22:6n3c, docosahexaenoic acid; C23:0, tricosylic; O/L, oleic/linoleic; SFA, saturated fatty acids; MUFA, monounsaturated fatty acids; PUFA, polyunsaturated fatty acids; AI, atherogenic index; TI, thrombogenic index).

For the oleic–linoleic acid ratio, the highest results were reported for almonds treated with 2 mmol L^−1^ KSi in both seasons. In 2023 and 2024, the application of both KSi treatments led to an increased ∑MUFA compared to the control, showing the highest values of this parameter in the 2023 season. The effect of the treatments on ∑PUFA was dependent on the KSi concentration; thus 20 mmol L^−1^ increased this parameter in both seasons, while 2 mmol L^−1^ reduced it in 2023. In 2024, the highest values of the PUFA/SFA ratio were observed in almonds treated with 20 mmol L^−1^ KSi and in the controls, while no significant differences were found in 2023. For both seasons, in almonds kernels, the ratio of PUFA to MUFA increased significantly with 20 mmol L^−1^ KSi treatment, while this treatment decreased (MUFA+PUFA)/SFA. No effect of 2 mmol L^−1^ KSi was found. In addition, significant (*P* < 0.05) differences were observed in the concentrations of margaric acid, oleic acid, *cis*‐vaccenic acid, linoleic acid, α‐linolenic acid, arachidonic acid, tricosylic acid, as well as in their MUFA and PUFA contents, when comparing the 2023 and 2024 seasons. No significant (*P* < 0.05) differences were found for the atherogenic and thrombogenic indexes with the treatments in any of the seasons. However, the thrombogenic index of almonds harvested in 2024 was significantly (*P* < 0.05) higher than those harvested in 2023.

## DISCUSSION

The application of inorganic salts has been widely used to extend the postharvest storage of fruits. These compounds have been classified as chemicals of low toxicity to human health and the environment. Studies concerning the use of inorganic salts, such as carbonates, sorbates, benzoates or silicates, have been focused on their ability to control the activity of fungal phytopathogens during postharvest fruit storage.^19^ However, previous results have highlighted that the application of solutions with K enhanced carbon assimilation, sugar transport and protein biosynthesis in plants,^11^ while silicon solutions increased photosynthesis by affecting chlorophyll concentration and the activities of RuBisCO and phosphoenolpyruvate (PEP)‐carboxylase enzyme for CO_2_ fixation.^20^ Preharvest strategies can alter the chemical composition of almonds, thereby affecting their quality traits, oil stability and mineral composition.^21^ Taking all this into account, the influence of preharvest treatments with KSi, which is considered to be a better stabilized Si and K source, on the quality parameters of almond kernels was evaluated.

The results showed that a 2 mmol L^−1^ KSi treatment had no effect, whereas a 20 mmol L^−1^ KSi treatment increased almond kernel weight. These results were consistent with previous results obtained for peaches^22^ and sweet peppers^13^ treated with foliar KSi. Texture parameters are important for the consumer acceptability. In this regard, the rupture strength is highly dependent on the moisture content, requiring greater strength at lower moisture content.^23^ However, our results showed no significant difference in moisture content when comparing the KSi‐treated almonds and controls, although a promising result was obtained with 20 mmol L^−1^ KSi, which increased almond hardness (Table 1). The L/W and sphericity provide important information about the shape of almond and are key to understanding its shelling behaviour, while GMD and surface area are related to the overall size of the almond and the surface area available for processes such as water absorption and kernel breakage.^24^ Almonds treated with 20 mmol L^−1^ KSi were more elongated than those treated with 2 mmol L^−1^ KSi or the controls, showing higher L/W values and lower sphericity values. Furthermore, the higher values of GMD and surface area were found in almonds treated with 20 mmol L^−1^ KSi, indicating larger kernels (Table 1). There is a general industry trend towards large kernels to facilitate and reduce the cost of cracking and blanching.^25^ The overall results showed, on the one hand, that the application of 20 mmol L^−1^ KSi was effective in improving some almond kernel quality parameters and, on the other hand, that the sizes of the almonds treated with 2 mmol L^−1^ KSi and the control group were smaller. However, all of the almonds met the minimum market requirements for commercialization.^26^

Previous results have demonstrated the significance of achieving a balanced nutrient profile in enhancing the quality of fruit by improving its physicochemical properties and bioactive compounds.^27^ It is well known that almonds are a healthy snack, as they are a good source of different compounds such as MUFA, protein, dietary fibre, vitamins and essential elements such as calcium, magnesium, phosphorus, potassium, zinc, copper and manganese.^28^ Therefore, it was relevant to evaluate the effect of KSi treatments on the nutritional composition of almonds. The interaction between KSi and nutrients of plants is complex and can be synergistic, neutral or antagonistic. In this regard, the results showed an effect of 2 mmol L^−1^ KSi concentration on P accumulation (Table 2). These results are consistent with those previously published for ‘Kinnow’ mandarins treated with foliar applications of K at a concentration of 100 mg L^−1^, which increased the phosphorus content of the juice.^29^ Potassium levels increased with both treatments, but the rise was more pronounced with the 2 mmol L^−1^ KSi application (Table 2). This finding aligns with previous soybean research, which has shown that increasing K rates does not always lead to significant (*P* > 0.05) increases in leaf or seed potassium content at various growth stages or at harvest maturity.^30^ Additionally, it has been suggested that the relationship between K application and tissue K levels can be complex and influenced by multiple factors.^31^ In this sense, the accumulation of K and P could be related to their easy phloem‐mobile capacity, which allows them to be translocated to the fruit.^32^ A generalized decrease in Mg was observed in KSi‐treated almonds (Table 2), which could be attributed to the cation competition between K and other divalent cations such as Mg.^33^ However, no effect of KSi on Ca concentration was observed. With regard to B content, the highest levels were found in almonds treated with KSi (Table 2). Recent results have indicated that, under normal or B‐deficient conditions, foliar KSi treatments could slightly increase B uptake, possibly indirectly through improved overall plant health.^34^ Thus, it is particularly important to increase the P and K content when consuming the recommended daily amount of almonds, since P is essential for energy production and bonds, while K regulates arterial pressure and muscle function.^35^

Among the health‐promoting compounds found in almonds, polyphenols can be highlighted.^36^ Our results showed a positive effect of the 20 mmol L^−1^ KSi treatment, which increased total antioxidant activity and total phenolic content in both seasons (Table 3). This effect could indicate that an excess of KSi produced a stress response in the fruit. In this sense, high K ion concentrations can modify the osmotic balance of the cell through ion transport and the induction of ROS.^37^ Consequently, the increase in TAA and TPC in almonds treated with 20 mmol L^−1^ KSi could be associated with an increase in ROS species. These results agree with those previously published for *Arabidopsis thaliana*, where high exposure to K was associated with physiological, metabolic and transcriptional changes that led to an increase in antioxidants and proline accumulation.^38^ Results for *Mentha spicata* showed that middle levels (325 mg L^−1^) of K improved plant growth, while a concentration in the range of 350–375 mg L^−1^ increased oxidative stress, inducing the accumulation of antioxidants and the activation of antioxidant enzymes.^39^ Furthermore, previous results on Valencia oranges with preharvest treatments with 20 mmol L^−1^ KSi showed the highest increase in TAA and TPC in the flavedo compared to those treated with 2 mmol L^−1^ KSi.^12^

The positive health effects of consuming almonds are mainly related to fatty acids, which contribute to enriching the diet by providing UFA. In this sense, MUFA intake has been shown to improve human health to a greater extent than PUFA intake, and obviously more than SFA.^40^ Moderate almond consumption (30 g per day) is an effective way to manage metabolic diseases by reducing low‐density lipoprotein and cholesterol and improving glycaemic control.^41^ Recent studies have concluded that almonds and their by‐products are suitable candidates for replacing some of the traditional fats included in the consumer's daily diet.^42^ Moreover, polar lipids are the main structural component in membranes, and changes in their fatty acid lipid composition directly affect membrane stability and function. Therefore, the fatty acid composition of almonds is used as an indicator of quality.^43^ The highest concentration of SFA was observed in almonds treated with 20 mmol L^−1^ KSi, which reduced the (MUFA + PUFA)/SFA ratio. There was no effect on almonds treated with 2 mmol L^−1^ KSi (Table 4). Previous studies have reported that foliar fertilization with K reduced the palmitic and stearic acid content of hazelnut cv. ‘Mortarella’, improving oil quality.^44^ The fact that in almonds treated with 20 mmol L^−1^ KSi the concentration of PUFA was higher than in the other samples could be a cause of worse oil stability during postharvest storage. This is because the presence of double bonds in the unsaturated fatty acids makes them susceptible to ROS compounds. The peroxidation of PUFA contributes to increasing the levels of ROS, reducing membrane stability, and promoting the accumulation of undesirable volatile compounds that negatively affect the sensory appeal of the fruit.^45^, ^46^ Oleic and linoleic acids are the main MUFA and PUFA, respectively, in almonds, and measuring their content provides information on fruit quality. High oleic acid content enhances oxidative stability, while high levels of linoleic acid promote almond spoilage.^47^ The highest oleic–linoleic acid ratios were found in almonds treated with 2 mmol L^−1^ KSi, while 20 mmol L^−1^ KSi did not have any effect on this ratio. These results could indicate high oxidative stability in almonds treated with 2 mmol L^−1^ KSi, which could prevent almonds from going rancid.^48^ Furthermore, it is important to highlight that 20 mmol L^−1^ KSi promoted the accumulation of both UFA, oleic and linoleic acids, which is consistent with a previous study on *Brassica napus* published by Shirani‐Rad *et al*. (2022),^49^ where the foliar application of KSi treatments elevated fatty acid unsaturation ratios.

Therefore, the practical implications of the KSi treatments for almond growers are significant, providing actionable strategies for enhancing profitability, product differentiation and sustainable farm management. The most direct benefit is the potential to increase the economic return due to the increase in weight, hardness and elongation observed in the 20 mmol L^−1^ KSi treatment. This could reduce postharvest losses related to physical damage caused by mechanical harvesting, handling, and processing, thus improving product quality. Furthermore, the increase in essential macronutrients and improvement in the fatty acid profile observed in almond kernels treated with 2 mmol L^−1^ KSi could be an advantage for marketing, as health‐conscious consumers may be willing to pay a premium for such products.

## CONCLUSIONS

The present study showed that the effect of preharvest treatments with KSi depended on the concentration. The most effective treatment for improving kernel weight, hardness and size was 20 mmol L^−1^ KSi. However, almonds treated with 2 mmol L^−1^ KSi showed a better antioxidant balance, fatty acid profile and nutrient composition. Furthermore, it was observed that almonds treated with 2 mmol L^−1^ KSi had the best oleic–linoleic acid ratio, which is essential for ensuring almond storability during postharvest, avoiding the development of undesirable flavours derived from lipid peroxidation. Regarding nutrients, the most effective treatment for increasing P, K and B content was 2 mmol L^−1^ KSi, while both concentrations reduced Mg concentration, and no effect was observed in Ca and Mn content. Therefore, KSi treatments have two potential applications in the almond industry depending on the applied concentration; 20 mmol L^−1^ KSi can be used to improve the desirable morphological and physical traits, while 2 mmol L^−1^ KSi can be used to enhance the nutritional composition without negatively affecting marketability.

## Data Availability

The data that support the findings of this study are available on request from the corresponding author. The data are not publicly available due to privacy or ethical restrictions.
